# Impact of incremental circumferential resection margin distance on overall survival and recurrence in oesophageal adenocarcinoma

**DOI:** 10.1002/bjs5.65

**Published:** 2018-04-23

**Authors:** W. R. C. Knight, J. Zylstra, W. Wulaningsih, M. Van Hemelrijck, D. Landau, N. Maisey, A. Gaya, C. R. Baker, J. A. Gossage, J. Largergren, A. R. Davies, the Guy's and St Thomas' Oesophago‐Gastric Research Group, M. Kelly, O. Hynes, G. Tham, C. Iezzi, A. Cowie, S. Ngan, A. Qureshi, M. Green, N. Griffin, A. Jacques, V. Goh, H. Deere, F. Chang, U. Mahadeva, B. Gill‐Barman, S. George, J. Dunn, S. Zeki, J. Meenan

**Affiliations:** ^1^ Department of Surgery, Guy's and St Thomas' Oesophago‐Gastric Centre, London, UK; ^2^ Department of Oncology, Guy's and St Thomas' Hospital, London, UK; ^3^ Division of Cancer Studies, King's College London, London, UK; ^4^ Cancer Epidemiology and Population Health Associated Research Group, King's College London, London, UK; ^5^ Medical Research Council Unit for Lifelong Health and Ageing, University College London, London, UK; ^6^ Upper Gastrointestinal Surgery, Department of Molecular Medicine and Surgery, Karolinska Institutet, Stockholm, Sweden; ^7^ Department of Surgery, Guy's and St Thomas' Oesophago‐Gastric Centre London UK; ^8^ Department of Oncology, Guy's and St Thomas Hospital London UK; ^9^ Department of Pathology, Guy's and St Thomas' Hospital London UK; ^10^ Department of Radiology, Guy's and St Thomas' Hospital London UK; ^11^ Cancer Imaging, School of Biomedical Engineering and Imaging Sciences, King's College London London UK; ^12^ Department of Cellular Pathology, Guy's and St Thomas' Hospital London UK; ^13^ Department of Gastroenterology, Guy's and St Thomas' Hospital London UK

## Abstract

**Background:**

Previous analyses of the oesophageal circumferential resection margin (CRM) have focused on the prognostic validity of two different definitions of a positive CRM, that of the College of American Pathologists (tumour at margin) and that of the Royal College of Pathologists (tumour within 1 mm). This study aimed to analyse the validity of these definitions and explore the risk of recurrence and survival with incremental tumour distances from the CRM.

**Methods:**

This cohort study included patients who underwent resection for adenocarcinoma of the oesophagus between 2000 and 2014. Kaplan–Meier and Cox regression analyses were performed to determine the hazard ratio (HR) with 95 per cent confidence intervals for recurrence and mortality in CRM increments: tumour at the cut margin, extending to within 0·1–0·9, 1·0–1·9, 2·0–4·9 mm, and 5·0 mm or more from the margin.

**Results:**

A total of 444 patients were included in the study. Kaplan–Meier and unadjusted analyses showed a significant incremental improvement in overall survival (P < 0·001) and recurrence (P for trend < 0·001) rates with increasing distance from the CRM. Tumour distance of 2·0 mm or more remained a significant predictor of survival on multivariable analysis (HR for risk of death 0·66, 95 per cent c.i. 0·44 to 1·00). Multivariable analysis of overall survival demonstrated a significant difference between a positive and negative CRM with the Royal College of Pathologists' definition (HR 1·37, 1·01 to 1·85), but not with the College of American Pathologists' definition (HR 1·22, 0·90 to 1·65).

**Conclusion:**

This study demonstrated an incremental improvement in survival and recurrence rates with increasing tumour distance from the CRM.

## Introduction

The introduction of neoadjuvant treatment has increased the survival of patients undergoing surgery for oesophageal cancer^1^, ^2^, ^3^. Despite this, 5‐year survival rates following resection rarely exceed 50 per cent, and recurrence rates are still disappointingly high. Understanding how various clinicopathological factors influence survival and patterns of recurrence may be important in guiding future tailored treatment strategies.

Many studies have examined the prognostic significance of the two most commonly used definitions of tumour‐involved circumferential resection margin (CRM). The College of American Pathologists (CAP) defines a positive CRM as tumour at the cut margin (TAM), which has been advocated in some studies^4^, ^5^, ^6^, ^7^, ^8^, ^9^, whereas the Royal College of Pathologists (RCP) defines a positive CRM as tumour within 1 mm, preferred in other studies^10^, ^11^, ^12^, ^13^. Some studies have found the CRM to be independently prognostically significant^4^ ^7^, ^9^ ^10^, ^13^, ^14^, ^15^, ^16^, and others have not^5^ ^6^, ^11^ ^12^, ^17^, ^18^, ^19^. A positive margin may increase the likelihood of locoregional and systemic tumour recurrence^9^ ^20^, although it is unclear whether the latter is simply a reflection of a larger, more advanced tumour.

The relationship between margin status and nodal status is already recognized as important. A study in 2006^15^ found that a positive CRM had greater prognostic significance in the presence of fewer nodal metastases. The presence of positive lymph nodes is known to confer a significantly worse prognosis^21^, so it may be that any independent prognostic significance of a positive CRM would be overshadowed by the presence of nodal disease.

This study aimed to evaluate the prognostic role of the two existing definitions of a positive CRM on overall survival and tumour recurrence in patients with oesophageal adenocarcinoma, and examine the influence of incremental increases in margin clearance on these outcomes, considering confounding factors such as nodal status.

## Methods

This was a cohort study using the database of consecutive resections performed at Guy's and St Thomas' Oesophago‐Gastric Centre, London, UK. The initial study cohort involved all patients who underwent oesophagectomy between 2000 and 2014. Only patients with adenocarcinoma who had undergone potentially curative oesophagectomy were included in the overall analysis. Patients with all other pathologies were excluded. The study exposure was CRM distance. For the analysis of incremental CRM distance, patients with a reported negative margin (no tumour within 1 mm) but with no documented CRM distance in millimetres, and patients who had a complete pathological response to chemotherapy, were excluded.

The primary outcome measures were overall all‐cause and disease‐specific mortality. Secondary outcomes were any recurrence, locoregional recurrence and systemic recurrence. Locoregional recurrence was defined as recurrent disease seen within the primary surgical or radiotherapy fields.

Patients underwent a standard protocol of investigation including oesophagogastroduodenoscopy, CT, endoscopic ultrasonography and fluorodeoxyglucose (FDG)‐PET. Neoadjuvant chemotherapy practice evolved during the study period, and followed standard indications and regimens as supported by RCT evidence^3^. The small number of patients who received neoadjuvant chemoradiotherapy were excluded. Patients judged to have an imaging status of T2 or above and/or lymph node positivity were considered for neoadjuvant chemotherapy if fit.

Transthoracic oesophagectomy included both Ivor Lewis and left thoracoabdominal approaches with two‐field lymphadenectomy^22^, ^23^, ^24^. Transhiatal oesophagectomy was performed in patients with lower oesophageal tumours in whom dissection of the primary could be achieved under direct vision from the abdomen along with an abdominal and lower mediastinal lymphadenectomy. Histological staging was standardized to meet the AJCC seventh edition TNM criteria. Pathological specimens were processed and reported using the RCP guidelines. The use of adjuvant chemotherapy or chemoradiotherapy was determined by multidisciplinary team consensus based on the positivity of resection margins, pathological nodal status and postoperative performance status of the patient.

### Statistical analysis

For overall all‐cause mortality, duration of follow‐up was defined as the time from surgery to the date of death or last follow‐up. For disease‐specific mortality, length of follow‐up was defined as the time from surgery to the date of recurrence or last date of follow‐up if disease‐free.

Kaplan–Meier curves were used to investigate crude differences in survival across different categories of CRM (TAM, 0·1–0·9, 1·0–1·9, 2·0–4·9 and 5·0 mm or more), which were tested formally using the log rank test. Analyses were stratified by: T category (T1–2 *versus* T3–4), neoadjuvant chemotherapy *versus* surgery alone, pathological lymph node involvement *versus* no such involvement, and presence of lymphovascular invasion *versus* no lymphovascular invasion. Cox regression was employed to obtain hazard ratios (HRs) and 95 per cent confidence intervals for time to death or recurrence based on categories of CRM. TAM was used as the reference category. Additional multivariable analysis was performed looking at TAM (reference category), 0·1–0·9, 1·0–1·9 and 2·0 mm or more to examine 1·0–1·9 mm as a possible ‘at risk’ group.

Further analysis compared the CAP and RCP definitions of CRM positivity with their corresponding negative groups used as the reference category to determine significance as an independent prognostic variable. In addition, a test for trend used CRM categories as a continuous variable. Findings were considered significant at *P* < 0·050 for both univariable and multivariable analyses.

The following clinicopathological parameters were adjusted for in the multivariable analyses: sex (male or female), age (continuous), preoperative stenting (yes or no), neoadjuvant chemotherapy (yes or no), type of surgery (transhiatal or transthoracic), lymphovascular invasion (yes or no), pT category (pT1–2 N0, pT1–2 N1, pT1–2 N2–3, pT3–4 N0, pT3–4 N1 or pT3–4 N2–3), pathological grade (poorly, moderately or well differentiated), Mandard tumour regression score (2–3, 4–5 or not applicable) and adjuvant treatment (none, chemotherapy or chemoradiotherapy). All analyses were conducted in R statistical software version 3·2.2 (R Foundation for Statistical Computing, Vienna, Austria).

## Results

The original cohort included 578 patients with oesophageal adenocarcinoma. The CRM positivity rate was 41·3 per cent (239 of 578) using RCP criteria, compared with 18·0 per cent (104 of 578) with CAP criteria. Rates of margin positivity reduced over time, and were 33·0 per cent (RCP) and 11·3 per cent (CAP) in the latter half of the study.

For the univariable and multivariable analyses of CRM distance, 22 patients with a complete pathological response to chemotherapy and 112 patients with reported negative margins (no tumour within 1 mm) but no CRM distance documented in millimetres were excluded. Histology specimens for this latter group were not available for retrospective histological analysis. Some 444 patients remained for final analysis, resulting in 104 patients in the TAM group, 135 in the 0·1–0·9‐mm group, 46 in the 1–1·9‐mm group, 64 in the 2·0–4·9‐mm group and 95 in the 5·0 mm or above group.

Patient characteristics are shown in *Table* 1. CRM‐positive patients (CAP and RCP) had a higher rate of other adverse prognostic factors, such as T3–4 status, poor differentiation, Mandard score 4 and 5, nodal disease and lymphovascular invasion (*Table* 1).

**Table 1:** Clinicopathological characteristics of patients with positive and negative resection margins, and according to resection margin increments

	Established margin definition			Specific margin difference (mm)			
	CAP CRM‐positive (TAM) (*n* = 104)	RCP CRM‐positive (< 1·0 mm) (*n* = 239)	CRM‐negative (≥ 1·0 mm) (*n* = 205)	0·1–0·99 (*n* = 135)	1–2 (*n* = 46)	2–5 (*n* = 64)	> 5 (*n* = 95)
Mean(s.d.) age at operation (years)	63·06(10·57)	62·17(9·58)	61·49(10·57)	61·49(8·72)	62·60(9·12)	63·64(10·49)	62·58(10·49)
Sex ratio (M : F)	84 : 20	196 : 43	185 : 20	113 : 22	40 : 6	56 : 8	89 : 6
Tumour location							
Siewert type 1	53 (51·0)	111 (46·4)	119 (58·0)	58 (43·0)	29 (63)	31 (48)	59 (62)
Siewert type 2	49 (47·1)	121 (50·6)	69 (33·7)	72 (53·3)	14 (30)	27 (42)	28 (29)
Lower oesophagus	2 (1·9)	7 (2·9)	17 (8·3)	5 (3·7)	3 (7)	6 (9)	8 (8)
Neoadjuvant chemotherapy							
Yes	76 (73·1)	191 (79·9)	150 (73·2)	115 (85·2)	41 (89)	52 (81)	57 (60)
No	28 (26·9)	48 (20·1)	55 (26·8)	20 (14·8)	5 (11)	12 (19)	38 (40)
Type of surgery							
TTO	51 (49·0)	135 (56·5)	90 (43·9)	82 (60·7)	23 (50)	24 (38)	43 (45)
THO	53 (51·0)	104 (43·5)	115 (56·1)	53 (39·3)	23 (50)	40 (63)	52 (55)
Pathological stage							
pT1–2 N−	3 (2·9)	11 (4·6)	82 (40·0)	8 (5·9)	7 (15)	19 (30)	56 (59)
pT1–2 N+	6 (5·8)	39 (16·3)	47 (22·9)	33 (24·4)	6 (13)	16 (25)	25 (26)
pT3–4 N−	18 (17·3)	32 (13·4)	33 (16·1)	14 (10·4)	10 (22)	14 (22)	9 (9)
pT3–4 N+	77 (74·0)	157 (65·7)	43 (21·0)	80 (59·3)	23 (50)	15 (23)	5 (5)
Pathological grade							
Poorly differentiated	58 (55·8)	124 (51·9)	64 (31·2)	66 (48·9)	12 (26)	23 (36)	29 (31)
Moderately differentiated	43 (41·3)	109 (45·6)	134 (65·4)	66 (48·9)	34 (74)	40 (63)	60 (63)
Well differentiated	4 (3·8)	6 (2·5)	7 (3·4)	3 (2·2)	0 (0)	1 (1)	6 (6)
Lymphovascular invasion							
Yes	75 (72·1)	172 (72·0)	80 (39·0)	97 (71·9)	26 (57)	33 (52)	21 (22)
No	29 (27·9)	67 (28·0)	125 (61·0)	38 (28·1)	20 (43)	31 (48)	74 (78)
Mandard score							
2–3 (good or partial response)	10 (9·6)	43 (18·0)	77 (37·6)	33 (24·4)	16 (35)	23 (36)	37 (39)
4–5 (poor or no response)	58 (55·8)	138 (57·7)	71 (34·6)	80 (59·3)	22 (48)	29 (45)	20 (21)
No chemotherapy	28 (26·9)	48 (20·1)	55 (26·8)	20 (14·8)	5 (11)	12 (19)	38 (40)
Not recorded	8 (7·7)	10 (4·2)	2 (1·0)	2 (1·5)	3 (7)	0 (0)	0 (0)
Adjuvant treatment							
None/not tolerated	35 (33·7)	82 (34·3)	117 (57·1)	47 (34·8)	23 (50)	33 (52)	61 (64)
Adjuvant chemotherapy	18 (17·3)	69 (28·9)	78 (38·0)	51 (37·8)	20 (43)	27 (42)	31 (33)
Adjuvant CRT	51 (49·0)	88 (36·8)	10 (4·9)	37 (27·4)	3 (7)	4 (6)	3 (3)
Recurrence							
None	35 (33·7)	92 (38·5)	125 (61·0)	57 (42·2)	21 (46)	41 (64)	63 (66)
Any	69 (66·3)	147 (61·5)	80 (39·0)	78 (57·8)	25 (54)	23 (36)	32 (34)
Local	32 (30·8)	75 (31·4)	47 (22·9)	43 (31·9)	15 (33)	11 (17)	21 (22)
Systemic	55 (52·9)	117 (49·0)	59 (28·8)	62 (45·9)	18 (39)	17 (27)	24 (25)

Values in parentheses are percentages unless indicated otherwise. CAP, College of American Pathologists; CRM, circumferential resection margin; TAM, tumour at the cut margin; RCP, Royal College of Pathologists; TTO, transthoracic oesophagectomy; THO, transhiatal oesophagectomy; CRT, chemoradiotherapy.

### Mortality

**Figure 1:**
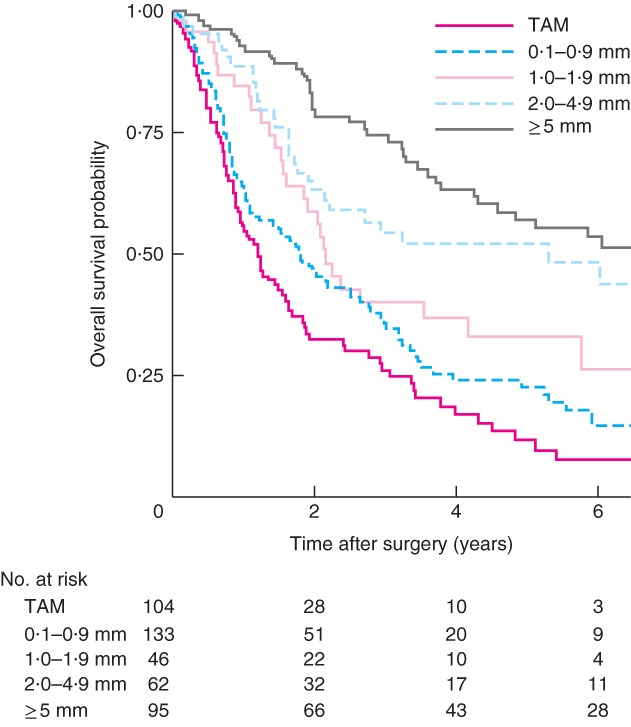
Kaplan–Meier curves of overall survival in patients who underwent resection of oesophageal adenocarcinoma, according to distance from the circumferential resection margin: tumour at the cut margin (TAM), 0·1–0·9‐mm, 1·0–1·9‐mm, 2·0–4·9‐mm and 5·0 mm and above groups. P < 0·001 (log rank test)

Kaplan–Meier analysis of overall survival in all patients demonstrated a survival advantage as tumour distance increased from the margin (*P* < 0·001) (*Fig*. 1). In unadjusted analysis, the overall all‐cause mortality decreased in increments away from the margin, with a significant improvement in survival between TAM and 0·1–0·9 mm (HR 0·71, 95 per cent c.i. 0·53 to 0·95), TAM and 1·0–1·9 mm (HR 0·47, 0·30 to 0·72), TAM and 2·0–4·9 mm (HR 0·34, 0·22 to 0·52) and TAM and 5·0 mm or more (HR 0·24, 0·17 to 0·36). This trend remained significant in multivariable analysis (*P* for trend < 0·050), although each category in isolation did not (*Table* 2).

**Table 2:** Unadjusted and multivariable Cox regression survival and recurrence analyses for the five groups with increasing distance from the resection margin

	Overall survival		Time to local recurrence		Time to systemic recurrence	
	Unadjusted	Multivariable	Unadjusted	Multivariable	Unadjusted	Multivariable
CRM distance (mm)						
TAM	1·00 (reference)	1·00 (reference)	1·00 (reference)	1·00 (reference)	1·00 (reference)	1·00 (reference)
0·1–0·9	0·71 (0·53, 0·95)	0·89 (0·65, 1·23)	0·95 (0·60, 1·50)	1·03 (0·62, 1·71)	0·80 (0·56, 1·15)	1·05 (0·71, 1·56)
1·0–1·9	0·47 (0·30, 0·72)	0·66 (0·44, 1·16)	0·80 (0·44, 1·49)	1·08 (0·54, 2·15)	0·56 (0·33, 0·96)	0·87 (0·49, 1·56)
2·0–4·9	0·34 (0·22, 0·52)	0·66 (0·41, 1·05)	0·37 (0·18, 0·73)	0·71 (0·33, 1·53)	0·34 (0·20, 0·59)	0·70 (0·38, 1·29)
≥ 5·0	0·24 (0·17, 0·36)	0·67 (0·41, 1·09)	0·35 (0·20, 0·61)	1·20 (0·58, 2·47)	0·25 (0·15, 0·41)	0·77 (0·41, 1·44)
*P* for trend	< 0·001	0·048	< 0·001	0·985	< 0·001	0·204

Values in parentheses are 95 per cent confidence intervals. CRM, circumferential resection margin; TAM, tumour at the cut edge.

### Recurrence

The local recurrence rate was 27·5 per cent (122 of 444): 30·8 per cent (32 of 104) in the TAM group, 31·9 per cent (43 of 135) in the 0·1–0·9‐mm group, 33 per cent (15 of 46) in the 1·0–1·9‐mm group, 17 per cent (11 of 64) in the 2·0–4·9‐mm group and 22 per cent (21 of 95) in the 5·0 mm or above group (*Table* 1). In unadjusted analysis, time to locoregional recurrence improved significantly only in the 2·0–4·9‐mm (HR 0·37, 95 per cent c.i. 0·18 to 0·73) and 5·0 mm or above (HR 0·35, 0·20 to 0·61) groups (*Table* 2). This difference was not significant in multivariable analysis.

The systemic recurrence rate was 39·6 per cent (176 of 444): 52·9 per cent (55 of 104) in the TAM group, 45·9 per cent (62 of 135) in the 0·1–0·9‐mm group, 39 per cent (18 of 46) in the 1·0–1·9‐mm group, 27 per cent (17 of 64) in the 2·0–4·9‐mm group and 25 per cent (24 of 95) in the 5·0 mm or above group. In unadjusted analysis, time to systemic recurrence improved significantly in the 1·0–1·9‐mm (HR 0·56, 95 per cent c.i. 0·33 to 0·96), 2·0–4·9‐mm (HR 0·34, 0·20 to 0·59) and the 5·0 mm or above (HR 0·25, 0·15 to 0·41) groups (*Table* 2). This difference was not significant in multivariable analysis.

### Margin definition: RCP versus CAP

Multivariable analysis of overall survival demonstrated a significant difference between a positive CRM and a negative CRM only with the RCP definition (RCP: HR 1·37, 95 per cent c.i. 1·01 to 1·85; CAP: HR 1·22, 0·90 to 1·65).

### Additional high‐risk group (1·0–1·9 mm)

Further analysis was performed to determine whether a 1·0–1·9‐mm CRM indicated a further ‘at risk group’ in addition to current definitions of a positive CRM. Regression analysis combining the 2·0–4·9 and 5·0 mm or above groups (2·0 mm or above group) indicated a significant difference in both overall and disease‐free survival when TAM, 0·1–0·9‐mm, 1·0–1·9‐mm and 2·0 mm or above groups were compared (*Table* 3). In multivariable analysis, the risk of death was significantly reduced when tumour was detected 2·0 mm or more from the cut margin *versus* TAM (HR 0·66, 95 per cent c.i. 0·44 to 1·00) (*Table* 3).

**Table 3:** Unadjusted and multivariable Cox regression survival and recurrence analysis for the four groups with increasing distance from the resection margin

	Overall survival	
	Unadjusted	Multivariable
CRM distance (mm)		
TAM	1·00 (reference)	1·00 (reference)
0·1–0·9	0·71 (0·53, 0·95)	0·89 (0·65, 1·23)
1·0–1·9	0·47 (0·30, 0·72)	0·66 (0·44, 1·16)
≥ 2·0	0·28 (0·20, 0·38)	0·66 (0·44, 1·00)
*P* for trend	< 0·001	0·045

Values in parentheses are 95 per cent confidence intervals. CRM, circumferential resection margin; TAM, tumour at the cut edge.

**Figure 2:**
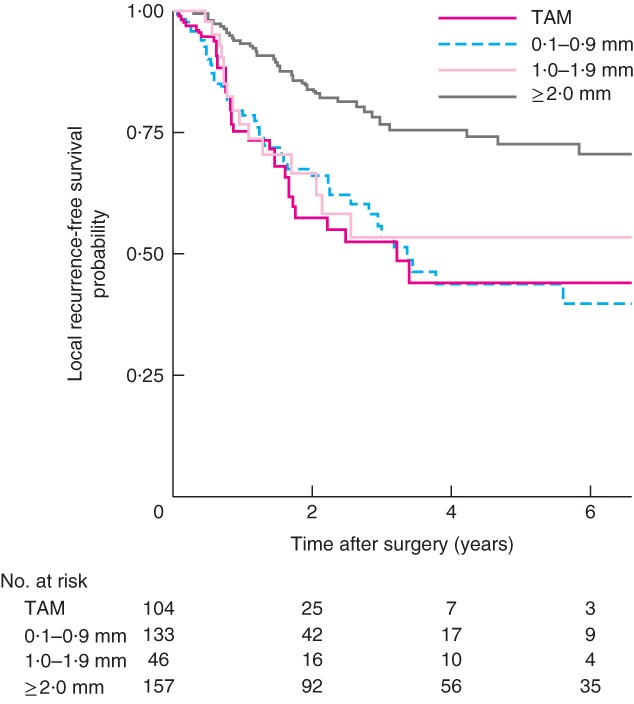
Kaplan–Meier curves of local recurrence‐free survival in patients who underwent resection of oesophageal adenocarcinoma, according to distance from the circumferential resection margin: tumour at the cut margin (TAM), 0·1–0·9‐mm, 1·0–1·9‐mm and 2·0 mm and above groups. P = 0·013 (2·0 mm and above versus TAM, log rank test)

**Figure 3:**
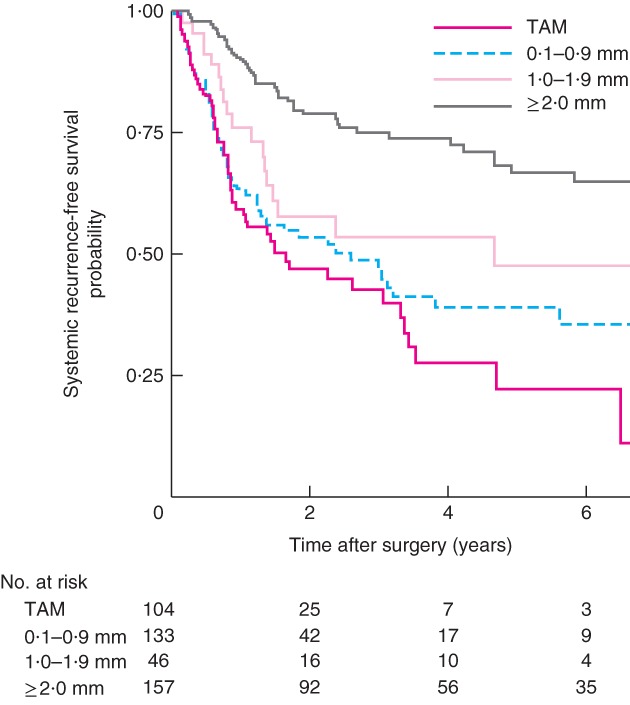
Kaplan–Meier curves of systemic recurrence‐free survival in patients who underwent resection of oesophageal adenocarcinoma, according to distance from the circumferential resection margin: tumour at the cut margin (TAM), 0·1–0·9‐mm, 1·0–1·9‐mm and 2·0 mm and above groups. P = 0·024 (2·0 mm and above versus TAM, log rank test)

Kaplan–Meier analysis of locoregional and systemic recurrence demonstrated a significant disease‐free survival benefit for a CRM of 2·0 mm or more when TAM, 0·1–0·9‐mm, 1·0–1·9‐mm and 2·0 mm or above groups were compared (*Figs* 2 and 3). The locoregional recurrence rate was lower in the 2·0 mm or above group at 20·8 per cent, compared with 31·6 per cent in the TAM group).

### Circumferential resection margin positivity in node‐negative patients

**Figure 4:**
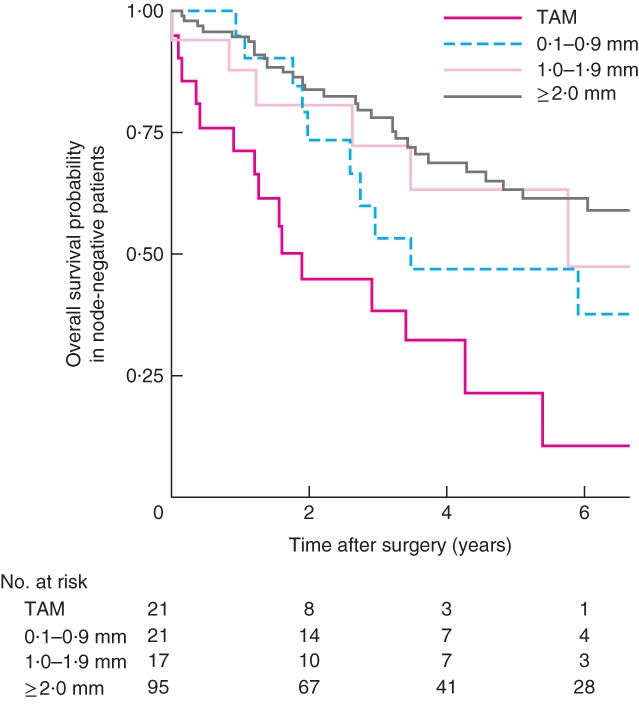
Kaplan–Meier curves of overall survival in node‐negative patients who underwent resection of oesophageal adenocarcinoma, according to distance from the circumferential resection margin: tumour at the cut margin (TAM), 0·1–0·9‐mm, 1·0–1·9‐mm and 2·0 mm and above groups. P = 0·041 (0·1–0·9 mm versus TAM, log rank test)

**Figure 5:**
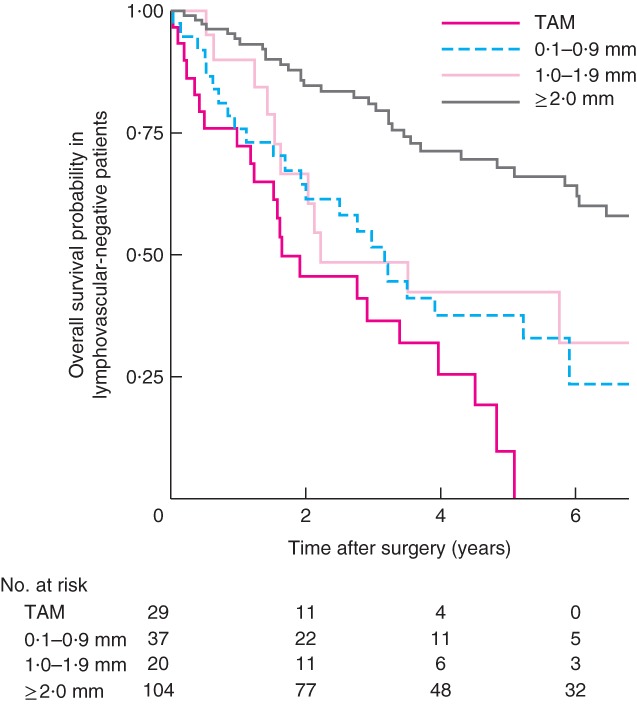
Kaplan–Meier curves of overall survival in lymphovascular‐negative patients who underwent resection of oesophageal adenocarcinoma, according to distance from the circumferential resection margin: tumour at the cut margin (TAM), 0·1–0·9‐mm, 1·0–1·9 mm and 2·0 mm and above groups. P = 0·074 (0·1–0·9 mm and 2·0 mm and above versus TAM, log rank test)

Kaplan–Meier analysis of TAM, 0·1–0·9‐mm, 1·0–1·9‐mm and 2·0 mm or above groups, stratified according to pathological node negativity, demonstrated a significant difference only between TAM and the 0·1–0·9‐mm cohort (*P* = 0·041) (*Fig*. 4). A similar pattern was observed in patients with no lymphovascular invasion, although the difference was not statistically significant (*Fig*. 5).

## Discussion

This study has demonstrated that overall survival improved with incremental increases in CRM distance following oesophagectomy for adenocarcinoma. Patients with tumours 1·0–1·9 mm from the resection margin had a significantly poorer prognosis than those with tumours with greater margins of clearance. This may represent an additional, as yet undescribed, ‘at risk’ group. This study found the RCP definition of CRM positivity to be independently prognostic.

Some methodological issues deserve attention. Analysing survival and recurrence of adenocarcinoma independent of squamous cell carcinoma reduced the heterogeneity of the cohort. Although comprehensive data collection allowed adjustment for confounders that affect recurrence and survival, the retrospective nature of the study and the evolution of practice throughout the study interval may have been additional sources of bias.

The prognostic value of the oesophageal CRM has been demonstrated in many studies^4^ ^8^, ^10^ ^11^, ^14^, ^15^, ^16^ ^25^, ^26^, ^27^, ^28^. A large multicentre study in 2016^9^ demonstrated that the CAP definition of CRM positivity was independently predictive of poor survival, whereas a meta‐analysis^29^ suggested that, although the CAP definition identified the highest‐risk group, the RCP definition encompassed patients still at risk of poor survival.

Some studies^6^ ^9^, ^16^ ^20^, ^30^ have examined the effect of CAP and RCP on rates and patterns of recurrence. A multicentre study^9^ (using the CAP definition of CRM positivity) found a significant increase in local recurrence rates between CRM‐positive and ‐negative patients (41·2 per cent *versus* 26·2 per cent respectively; *P* < 0·001) after propensity score matching, but no difference in systemic recurrence rates (28·3 *versus* 28·9 per cent; *P* = 0·664). Another study^20^ (using the RCP definition) found no increase in locoregional recurrence in CRM‐positive patients (HR 0·7, 95 per cent c.i. 0·3 to 2·1) but an increased risk of systemic recurrence (HR 3·0, 1·5 to 5·9).

Locoregional recurrence rates were similar in the TAM, 0·1–0·9‐mm and 1·0–1·9‐mm groups in the present study. In unadjusted analysis there was a significant reduction in the risk of local recurrence between the 1·0–1·9‐mm group and 2·0 mm or above group, with the overall trend reaching significance. The lack of significance in multivariable analysis may be because patients with positive margins were offered adjuvant chemoradiotherapy or as a result of unmeasured confounders. As the study showed similar locoregional recurrence rates in TAM, 0·1–0·9‐mm and 1·0–1·9‐mm groups, the mechanism by which CRM distance impacts on survival may be more complicated than inadequate local tumour clearance.

Survival analysis of patients with node‐negative disease found the CAP definition (tumour at margin) to confer a significantly worse outcome in comparison with patients with no tumour at the margin, a relationship that was not found in node‐positive patients. It has long been known that pathological nodal status is one of the most important prognostic markers of adenocarcinoma of the oesophagus^21^. It would follow that the independence of the CRM as a prognostic marker might diminish with lymph node metastases, given the relationship between lymph node metastasis and poor outcome. A number of studies^15^ ^16^, ^31^ have stratified node‐positive patients when analysing the impact of CRM positivity on survival, but with mixed results. One study^15^ showed that the RCP definition of CRM was adversely prognostic only when less than 25 per cent of the lymph node yield was positive. A meta‐analysis^29^ concluded that the presence of lymph node metastasis appeared to negate the importance of CRM involvement. This suggests that the focus of treatment of patients with nodal disease should be effective systemic control.

In the present study, patients with tumour within 1·0–1·9 mm of the CRM had an independent significant risk of worse prognosis. This was reflected in the disease‐free survival curves, where the only significant difference between curves was seen between the 1·0–1·9‐mm group and the 2·0 mm or above group. Tumour within 1·0–1·9 mm of the CRM seems to represent a prognostic ‘middle ground’ in terms of both survival and recurrence. This may be important when considering adjuvant treatment strategies.

Given the stepwise improvement in survival as tumour is found further away from the margin, it may be more helpful to consider CRM distance as a continuous parameter rather than a positive/negative phenomenon.

## Collaborators

Other members of the Guy's and St Thomas' Oesophago‐Gastric Research Group are: M. Kelly, O. Hynes, G. Tham, C. Iezzi and A. Cowie (Department of Surgery, Guy's and St Thomas' Oesophago‐Gastric Centre, London, UK); S. Ngan and A. Qureshi (Department of Oncology, Guy's and St Thomas Hospital, London, UK); M. Green (Department of Pathology, Guy's and St Thomas' Hospital, London, UK); N. Griffin and A. Jacques (Department of Radiology, Guy's and St Thomas' Hospital, London, UK); V. Goh (Cancer Imaging, School of Biomedical Engineering and Imaging Sciences, King's College London, London, UK); H. Deere, F. Chang, U. Mahadeva, B. Gill‐Barman and S. George (Department of Cellular Pathology, Guy's and St Thomas' Hospital, London, UK); J. Dunn, S. Zeki and J. Meenan (Department of Gastroenterology, Guy's and St Thomas' Hospital, London, UK).
